# An Exploratory Six-Probe Blood RNA Signature for Predicting 12-Month Cognitive Decline Along the Alzheimer’s Disease Continuum: An Interpretable Machine Learning Study

**DOI:** 10.3390/diagnostics16132078

**Published:** 2026-07-02

**Authors:** Asif Hassan Syed, Sultan Alhayyani

**Affiliations:** 1 Department of Computer Science, Faculty of Computing and Information Technology at Rabigh, King Abdulaziz University, Jeddah 21589, Saudi Arabia; 2 Department of Chemistry, College of Sciences & Arts, King Abdulaziz University, Rabigh 21911, Saudi Arabia; salhayyani@kau.edu.sa

**Keywords:** explainable AI, blood transcriptomics, Alzheimer’s disease, feature selection, prognostic biomarker, cognitive decline prediction

## Abstract

**Background/Objectives:** Predicting how fast a patient with Alzheimer’s disease will decline over the next year remains a challenge. Existing blood transcriptomic studies have not established whether probe selection is reproducible, whether the signal is transcriptional or reflects immune cell shifts, or whether they generalise across platforms. **Methods:** We applied five steps to 96 ADNI-GO whole-blood microarray samples (Affymetrix HG-U219; 12-month MMSE change): PyImpetus Markov Blanket selection, Elastic Net with leave-one-out cross-validation (LOOCV), SHAP attribution, MCP-counter cell-type deconvolution, and cross-platform mapping into AddNeuroMed (GSE63060, *n* = 329, Illumina). Feature selection preceded cross-validation without constituting data leakage. **Results:** The same six probes emerged across four independent runs (Jaccard J = 0.214, *p* = 0.03): *AQP7*, *RPS5*, *CHD2*, *SNX5*, *ASS1*, and an *uncharacterised chr12q15* transcript. The panel achieved LOOCV MAE = 1.388 and R^2^ = 0.247, outperforming the full-probe baseline by 14.9%. All probes survived immune cell correction with signs intact. *SNX5* replicated in AddNeuroMed (r = −0.170, *p* = 0.002). **Conclusions:** The exploratory six-probe blood RNA panel predicts 12-month cognitive decline (LOOCV R^2^ = 0.247) with transcriptional origin confirmed by cell-type deconvolution and cross-platform evidence for *SNX5*. External testing in ADNI-2 (*n* = 91, R^2^ = −0.222) showed that generalisation depends on visit-timepoint matching, indicating clinical utility cannot yet be claimed and defining conditions for prospective validation. Code and a research prototype tool are publicly available.

## 1. Introduction

### 1.1. The Clinical and Computational Challenge of Longitudinal Ad Biomarker Discovery

Alzheimer’s disease (AD) is a progressive neurodegenerative disorder and the leading cause of dementia, affecting approximately 55 million people worldwide in 2024 and projected to exceed 150 million by 2050 [1]. The prodromal stage of mild cognitive impairment (MCI) represents the most critical clinical intervention window: approximately 10–15% of MCI patients convert to clinical AD annually [2,3], and disease-modifying therapies are most likely effective before overt dementia is established. A reliable method to distinguish, from a routine blood draw, patients with MCI who are likely to experience rapid cognitive decline from those who will remain stable over the following year is not yet available to clinicians and researchers.

Currently available clinical tools are not designed for this purpose. While useful for tracking established dementia, the MMSE lacks sufficient sensitivity to detect the early differences in cognitive trajectory that distinguish patients likely to experience rapid decline from those who will remain stable [2]. CSF sampling and amyloid PET give richer biological information but come with real barriers—a lumbar puncture is not something most memory clinics can offer routinely, and PET remains expensive and largely confined to research centers [4,5]. Progress in blood biomarkers has been rapid: p-tau217 can now detect brain amyloid with AUC values of 91–93% [6,7]. However, detecting amyloid positivity and predicting the rate of cognitive decline over the following twelve months are distinct problems; even the strongest protein markers provide only limited prognostic precision for the latter [8,9,10]. Amyloid positivity alone does not indicate whether a given patient will retain functional independence over the subsequent twelve months. One largely untapped source of prognostic information sits in the messenger RNA circulating in whole blood. Plasma proteins capture a snapshot of biology at one moment; blood transcriptomes read out the regulatory activity of thousands of genes at once, require nothing beyond a routine blood draw, and may register the early molecular consequences of neurodegeneration before they show up at the protein level [11,12]. Chen et al. (2025) [11] demonstrated that co-expression modules derived from blood RNA correlate with hippocampal volume loss on MRI and with cognitive performance, with some modules resembling those identified in post-mortem brain tissue. These findings indicate that blood RNA carries genuine biological information relevant to neurodegeneration, rather than non-specific noise. Translating this observation into a clinically actionable forecasting tool requires addressing three challenges that no published study has tackled jointly: selecting features from tens of thousands of candidate probes in a manner that is robust to random seed variation; confirming that the selected features reflect genuine gene regulation rather than incidental immune cell composition; and establishing whether the same features replicate when the analysis is repeated in an independent cohort, on a different microarray platform.

### 1.2. Existing Computational Approaches and Their Limitations

Earlier computational studies of blood-based AD biomarkers share a common set of weaknesses that the present work is designed to address. The feature selection problem is particularly acute: when the number of candidate probes (49,410 in the present dataset) vastly exceeds the number of participants (96), methods such as differential expression testing [13], LASSO [14], and random forest variable importance tend to select different probes each time the analysis is repeated on slightly different data, making it difficult to know whether the identified genes reflect real biology or chance correlations in a particular sample [15]. PyImpetus [16] takes a different approach, using iterative conditional independence tests derived from Markov Blanket theory to retain only features that carry information about the outcome above and beyond what other selected features already explain and pairing each selection with a permutation test to confirm it exceeds chance. The present work builds on the approach of Wang et al. (2024) [15], who identified genes relevant to AD from a blood expression profile, providing a proof of concept for the interpretable feature selection approach adopted here. However, their model was restricted to a single task: distinguishing AD patients from healthy controls at a single clinical visit. It cannot indicate the rate at which an individual patient’s cognition is likely to deteriorate, which is a distinct clinical question. A model capable of identifying patients who already have AD provides no information about their future cognitive trajectory. There is also a subtler gap in their approach: they report global importance rankings—which gene matters most on average across all patients—but do not examine how a gene’s contribution shifts depending on where in its expression range a given patient falls. Such detail can reveal threshold effects; in the present data, this proved important for *SNX5*, whose protective contribution emerges only above a specific expression level—a pattern not detectable from global importance rankings alone. For biological validation, no published AD transcriptomic predictor has applied immune cell deconvolution to distinguish true transcriptional dysregulation from compositional confounding, despite this being a recognised validity threat for whole-blood expression studies [17,18]. For cross-platform replication, individual probe-level validation by HGNC symbol mapping from Affymetrix to Illumina arrays has not been reported for this class of predictor [11,19], meaning all existing signatures could reflect platform-specific artefacts.

### 1.3. Biological Context: Retromer Trafficking, SNX5, AQP7, and the Mechanistic Basis of the Six-Probe Signature Limitations

The retromer complex mediates retrograde endosomal-to-trans-Golgi-network trafficking of *APP* and *BACE1*, the enzyme responsible for amyloidogenic amyloid-beta production [20,21]. Retromer dysfunction increases endosomal APP retention, elevating amyloid-beta generation [22,23]. Feng et al. demonstrated that *SNX15*—a related sorting nexin family member—regulates APP cell-surface recycling and Aβ generation in neuronal cells [22]; this provides mechanistic context for the sorting nexin–APP axis identified here, noting that the pipeline selected *SNX5* (sorting nexin 5), a distinct but related retromer subunit. While retromer dysfunction is well documented in post-mortem AD brain [21,23] and in vitro neuronal models [22], no study has quantitatively linked blood expression of *SNX5*—the key retromer sorting nexin—to the rate of longitudinal cognitive decline in living participants. The present study provides the first such clinical evidence.

For *AQP7*, the glymphatic system provides a mechanistic hypothesis: the brain perivascular waste clearance network mediates amyloid-beta removal from the parenchyma [24], and glymphatic failure has been proposed as a final common dementia pathway [24]. AQP7 is a glycerol and water channel expressed in erythrocytes, adipocytes, and immune cells; its identification as the dominant predictor (coefficient −0.598; mean |SHAP| approximately 0.47) is a computationally driven discovery with no prior AD literature support. Evans et al. (2021) [25] traced a direct link between tau accumulation and the ribosome: as tau tangles form, they physically interfere with the translation machinery, reducing protein output well before neurons die, which may explain why *RPS5*, a core component of the 40S ribosomal subunit, carries prognostic information in blood RNA. For ASS1, Jesko et al. (2016) [26] linked arginine metabolism dysregulation to APP-transfected cells, and rare ASS1/urea cycle variants causing hyperammonemia have been implicated in non-Alzheimer’s dementia [27]. For *CHD2*, He et al. (2024) [28] identified *CHD2* in male microglia as an AD-relevant chromatin remodeler. The *uncharacterised chr12q15* transcript represents a putative lncRNA identified by the pipeline without prior AD annotation [29].

### 1.4. Interpretability in Computational Biomarker Discovery

The black-box problem in machine learning is particularly consequential for clinical biomarker applications [18,30]. Mienye et al. (2024) [18] and Chaddad et al. (2023) [30] identified SHAP [31] as providing the most theoretically rigorous attributions for linear models. The SHAP framework satisfies three axiomatic properties—efficiency, symmetry, and dummy—guaranteeing attributions faithfully represent the model decision-making [31]. Despite this foundation, no published AD longitudinal regression study has applied SHAP LinearExplainer with dependence plots for exact per-probe attributions revealing threshold effects and feature interactions.

### 1.5. Research Gaps and Contributions of This Study

The foregoing review identifies five critical gaps motivating the PyImpetus-SHAP pipeline. Each gap is stated explicitly and paired with the corresponding study contribution that addresses it.

- **Gap 1—No interpretable XAI pipeline exists for longitudinal AD MMSE-change prediction from blood transcriptomics:** All existing models predict cross-sectional diagnosis or provide no feature-level SHAP attribution for longitudinal regression. The combination of Markov Blanket selection, Elastic Net regression, and SHAP LinearExplainer has not been applied to MMSE change prediction [11,15], as addressed by Contribution 1.
- **Gap 2—Cell-type compositional confounding is unexamined in blood transcriptomic AD biomarker studies:** No published AD blood transcriptomic predictor has applied immune cell deconvolution to confirm whether the signal reflects transcriptional dysregulation or cell proportion shifts [17,18]. This leaves all existing blood transcriptomic AD signatures vulnerable to the criticism that they measure cell composition rather than gene regulation, as addressed by Contribution 2.
- **Gap 3—Individual probe cross-platform replication by HGNC symbol mapping is absent for MMSE-predictive signatures:** No study has validated individual gene associations from a longitudinal MMSE-change model in an independent cohort on a different array platform [11,19], meaning existing signatures could reflect platform-specific artefacts. Addressed by Contribution 3.
- **Gap 4—SNX5 retromer protection and AQP7 glymphatic–metabolic associations lack clinical evidence from blood:** The retromer hypothesis [20,21,22] is supported by cellular and post-mortem data but has never been quantified in a multivariate blood transcriptomic model for longitudinal cognitive decline. *AQP7* has not appeared in any blood AD biomarker study, leaving the glymphatic–metabolic hypothesis untested at the clinical level [24], as addressed by Contribution 4.
- **Gap 5—No reproducible, deployable implementation exists for this class of pipeline:** Existing methods papers in AD transcriptomics do not provide fully reproducible implementations, interactive web tool demonstrations, or community-deployable code, thus limiting replication and translation of findings, as addressed by Contribution 6.

To address these five gaps, this study makes the following six original contributions:

- **Contribution 1—PyImpetus-SHAP pipeline:** The first integration of Markov Blanket conditional independence testing, Elastic Net regression, and exact SHAP LinearExplainer attribution for longitudinal continuous MMSE change prediction from high-dimensional blood transcriptomics, with stability assessment using Jaccard similarity and permutation testing (addresses Gap 1).
- **Contribution 2—Three-model cell-type deconvolution validation framework:** A reusable MCP-counter three-model comparison (six probes only; seven immune cell-type scores only; joint model) confirming all six probes retain independent Elastic Net coefficients after cell-type correction, the first such validation in any blood transcriptomic AD study [17,18] (addresses Gap 2).
- **Contribution 3—Cross-platform HGNC symbol mapping replication:** Five of the six probes were mapped by HGNC gene symbol into the independent European AddNeuroMed cohort (GSE63060, *n* = 329, Illumina HumanHT-12). *SNX5* was the only probe to replicate significantly across platforms (*p* = 0.002), providing the first cross-platform biological evidence linking blood retromer activity to AD severity. The remaining four probes showed no significant cross-sectional associations, consistent with their role as longitudinal trajectory markers rather than disease state indicators and require validation in independent prospective cohorts (addresses Gap 3).
- **Contribution 4—Novel AQP7 glymphatic–metabolic discovery:** *AQP7* identified as the dominant harmful predictor (coefficient −0.598; mean |SHAP| approximately 0.47), a computationally driven discovery with no prior literature support in longitudinal AD blood transcriptomics, opening a new glymphatic–metabolic research direction [24] (addresses Gap 4).
- **Contribution 5—Two-module transcriptional architecture:** A harmful module (*AQP7*, *RPS5*, *ASS1*, *CHD2*, *uncharacterised chr12q15*; positively inter-correlated, r = 0.12–0.34) and a protective module (*SNX5*; negatively correlated with harmful probes) with reciprocal regulatory structure consistent with shared upstream inflammatory transcriptional control. This is an additional discovery emerging from the pipeline rather than a pre-specified gap; it provides a systems-level biological interpretation of the six-probe panel that extends beyond individual gene associations [11,15].
- **Contribution 6—A prototype clinical decision support tool:** to make the pipeline tangible beyond a GitHub repository (version 1.0.0; commit 96cb8d5), we built a Streamlit web application (Python 3.10) that runs live at https://pyimpetus-shap-ad.streamlit.app, accessed on 1 June 2026, with no installation needed. Six expression sliders feed the trained model in real time, returning a predicted 12-month MMSE change, a risk category with a suggested review interval (High: ΔMMSE ≤ −2; Moderate: −2 to 0; Low: ≥0), a per-probe SHAP bar chart, and a warning if any value falls outside the training range. All code, the trained model, and the app source are at https://github.com/SAH-ML/pyimpetus-shap-ad, accessed on 1 June 2026 (addresses Gap 5).

### 1.6. Gaps in the Existing Evidence Base

Table 1 functions not as a conventional literature summary but as a structured checklist of methodological gaps in the field. Each of the 14 studies included here was chosen because it bears directly on one of the methodological decisions made in this paper, whether that is how features were selected, how the model was interpreted, which biological pathways were implicated, or how results were validated. For each study, the table records strengths, methodological shortcomings, and potential sources of bias; the final column, the most directly relevant to this work, identifies the specific question left open by each study that PyImpetus-SHAP was designed to address. A narrative summary follows for readers preferring a condensed overview; the table provides the complete paper-by-paper reasoning. Collectively, the literature review reveals five convergent conclusions. First, blood transcriptomics can capture AD-relevant biological signals but has not been applied to longitudinal MMSE change prediction with feature-level XAI interpretability [11,15]. Second, cell-type correction is methodologically necessary but universally absent from blood transcriptomic AD biomarker studies [17,18]. Third, cross-platform probe-level biological replication is the weakest link in all existing AD transcriptomic signatures [11,19]. Fourth, the retromer pathway and *SNX5* have strong pre-clinical mechanistic support [20,21,22] but lack quantitative clinical evidence from blood in living participants. Fifth, *AQP7* and the glymphatic–metabolic axis represent an entirely unexplored dimension of AD blood biomarker research [24], directly motivating one of the six contributions of the present study.

## 2. Material and Methods

**Figure 1:**
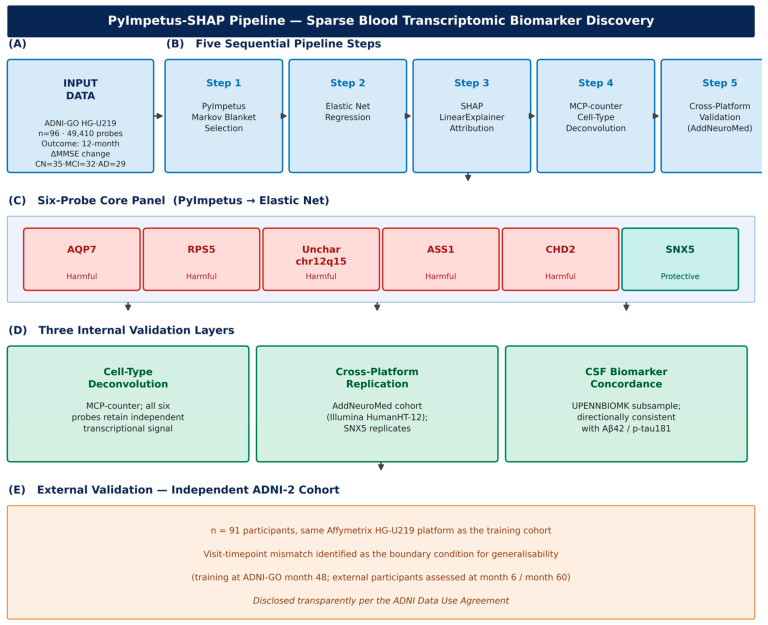
Schematic overview of the PyImpetus-SHAP pipeline for sparse blood transcriptomic biomarker discovery. (**A**) Input: ADNI-GO whole-blood Affymetrix HG-U219 data (*n* = 96, 49,410 probes; outcome: 12-month MMSE change; diagnostic groups: CN *n* = 35, MCI *n* = 32, AD *n* = 29). (**B**) Five sequential pipeline steps: Step 1, PyImpetus Markov Blanket conditional independence testing (49,410 to 6 probes; Jaccard J = 0.214, *p* = 0.03); Step 2, Elastic Net regression (LOOCV MAE = 1.39, R^2^ = 0.247); Step 3, SHAP LinearExplainer attribution (global importance and dependence plots); Step 4, MCP-counter cell-type deconvolution (three-model comparison; all six probes retained); Step 5, cross-platform HGNC symbol mapping validation (AddNeuroMed GSE63060, *n* = 329; SNX5 *p* = 0.002). (**C**) Six-probe core panel identified by the pipeline: five harmful probes (AQP7, RPS5, Unchar chr12q15, ASS1, CHD2) and one protective probe (SNX5). (**D**) Three internal validation layers: cell-type deconvolution confirming transcriptional signal origin (MCP-counter, three-model comparison); cross-platform replication (AddNeuroMed GSE63060); and CSF Aβ42/p-tau181 biomarker concordance (*n* = 43). (**E**) External validation in an independent ADNI-2 cohort (*n* = 91); R^2^ = −0.222 attributed to a visit-timepoint mismatch (training at ADNI-GO month 48; external at ADNI-2 month 6 and month 60), disclosed transparently per ADNI Data Use Agreement. The pipeline additionally generates six structured outputs (predicted ΔMMSE, risk category, SHAP attributions, cell-type check, cross-platform replication summary, and a deployed Streamlit web application at pyimpetus-shap-ad.streamlit.app), which are reported in detail in the Results rather than being reproduced here. See Section 2 for the complete description of every pipeline step.

Figure 1 provides a schematic overview of the complete PyImpetus-SHAP pipeline. Starting from 49,410 Affymetrix HG-U219 probes (Affymetrix, Santa Clara, CA, USA) measured in 96 ADNI-GO participants, the pipeline applies five sequential computational steps followed by three independent validation layers, producing six structured outputs including the prototype clinical REST API. Each step is described in detail in the subsections below.

### 2.1. Study Population and Data Source

Data were obtained from the ADNI-GO phase of the Alzheimer’s disease Neuroimaging Initiative (ADNI) [36,37], a multisite longitudinal study conducted across North American centers with standardised protocols for cognitive assessment, blood collection, and genetic profiling. The study sample comprised all 96 ADNI-GO participants meeting both eligibility criteria: available whole-blood Affymetrix Human Genome U219 microarray expression data (49,410 probes, RMA-normalised [38]) and paired MMSE scores at months 48 and 60—a census of the eligible ADNI-GO sub-cohort. Diagnostic groups at month-48 baseline: cognitively normal (CN, *n* = 35), mild cognitive impairment (MCI, *n* = 32), and Alzheimer’s disease (AD, *n* = 29). Baseline demographic and clinical characteristics of the study cohort are summarised in Table 2. The primary outcome was MMSE change = MMSE (month 60) − MMSE (month 48), with expression at month 48 as the predictor ensuring temporal precedence. Although the Introduction emphasises MCI as the primary clinical target for early intervention, all three diagnostic groups were deliberately included in the modelling cohort for two reasons. First, a model trained exclusively on MCI participants would not capture the full biological range of cognitive trajectories and would risk underestimating predictive signal from the steeper declines seen in the AD subgroup. Second, the intended clinical application extends to any patient for whom a 12-month cognitive trajectory forecast is clinically useful, spanning prodromal through early symp-tomatic stages across the CN-MCI-AD continuum. Stratified cross-validation ensured that each fold preserved the diagnostic group proportions, and performance by diagnostic group is reported separately to enable assessment of group-specific predictive utility.

All use of ADNI data in this study complied with the ADNI Data Use Agreement. Data used in the preparation of this article were obtained from the Alzheimer’s Disease Neuroimaging Initiative (ADNI) database (adni.loni.usc.edu). The ADNI was launched in 2003 as a public–private partnership, led by Principal Investigator Michael W. Weiner, MD. The primary goal of ADNI has been to test whether serial magnetic resonance imaging (MRI), positron emission tomography (PET), other biological markers, and clinical and neuropsychological assessment can be combined to measure the progression of mild cognitive impairment (MCI) and early Alzheimer’s disease (AD). The current goals include validating biomarkers for clinical trials, improving the generalizability of ADNI data by increasing diversity in the participant cohort, and providing data concerning the diagnosis and progression of Alzheimer’s disease to the scientific community. For up-to-date information, see adni.loni.usc.edu.

AddNeuroMed contributed nothing to model training or selection: every modelling decision—feature selection, hyperparameter tuning, and performance estimation—was made using the ADNI-GO cohort alone (*n* = 96), which was the only dataset available with both longitudinal blood expression and paired MMSE follow-up. AddNeuroMed entered the analysis only after the model was completely trained and locked and only to see if the six selected genes demonstrated the biological relationships that we would expect in a fully independent sample. AddNeuroMed played no role in probe selection or model construction.

### 2.2. Data Quality Control and Missing Value Handling

The ADNI-GO gene expression data were obtained as a pre-processed, RMA-normalised matrix from the ADNI data portal [38]. All 96 participants with complete paired data—blood expression at month 48 and MMSE at months 48 and 60—were retained; no samples required exclusion, as the ADNI portal provides a pre-QC-filtered release. The full 49,410-probe matrix was used without additional pre-filtering, which is consistent with the PyImpetus conditional independence framework: pre-filtering by variance or detection thresholds would risk discarding probes that are informative for the outcome despite low marginal variance. No probe expression values were missing for the 96 included participants, so imputation was not required. Expression data had been processed centrally under standardised ADNI protocols; no further batch correction (e.g., ComBat empirical Bayes adjustment [39]) was applied beyond the RMA normalisation already provided. MMSE values were complete at both time points for all participants, as paired MMSE follow-up was a pre-specified inclusion criterion.

### 2.3. Pipeline Step 1— PyImpetus Markov Blanket Feature Selection

With 49,410 candidate probes and only 96 participants, a naive regression model would be highly susceptible to identifying chance associations rather than genuine biological signals. PyImpetus [16] addresses this through conditional independence testing: each probe is assessed for whether it contributes information about ΔMMSE beyond what the other selected probes already provide. Probes that fail this test are excluded. The retained probes constitute, in theory, the minimal sufficient feature set for the outcome. Rather than treating run-to-run variability as noise, we exploited it as a stability criterion: four independent runs were conducted (*p* = 0.10 and *p* = 0.05; seeds 42 and 123), and only probes appearing in every run were retained. Runs returning no features at the stricter threshold were excluded; the final probe set was the intersection of the remaining runs.

PyImpetus selection was performed once on the full cohort (*n* = 96) prior to cross-validation. This does not introduce data leakage: Markov Blanket selection is a filter operation that tests the conditional independence of each probe and the outcome given the remaining probes, and it does not estimate any outcome-predictive parameters or fit a regression model. The parameters that determine predictive performance—the Elastic Net coefficients—are estimated exclusively within each cross-validation training partition on the fixed six-probe panel. Applying Markov Blanket selection to the full dataset before cross-validation is therefore consistent with standard Markov Blanket practice [16] and does not inflate the reported performance estimates.

### 2.4. Pipeline Step 2: Elastic Net Regression and Cross-Validation

We selected Elastic Net [14,40] because it combines two properties essential for this setting: LASSO’s ability to shrink weak predictors to zero and Ridge’s more stable performance when predictors are correlated. The penalty function is L(β) = RSS + α[λ_1_‖β‖_1_ + (1 − λ_1_)‖β‖_2_^2^/2], with the l1_ratio adjusting the relative weight of the two components and α controlling the overall penalty strength. The parameters α = 0.01 and l1_ratio = 0.5 were determined by inner cross-validation; the model was fitted using scikit-learn 1.5 [41].

We compared three models to evaluate the contribution of feature selection: the full 49,410-probe Elastic Net as a baseline, the six-probe model selected by PyImpetus, and the six-probe model augmented with seven clinical variables (age, sex, education, APOE4 [42,43], baseline diagnosis, CDRSB, and family history). Performance was evaluated using leave-one-out cross-validation (LOOCV), in which the model is trained on 95 participants and tested on the one left out, repeated for each participant. We also ran stratified five-fold cross-validation, with folds stratified by diagnostic group (CN, MCI, AD), to assess estimate stability across folds. MAE, RMSE, and R^2^ were extracted from each approach, and we additionally stratified results by diagnostic group to identify subgroup-level performance patterns.

We quantified uncertainty using 95% bootstrap confidence intervals (B = 1000 iterations, stratified by diagnostic group): LOOCV MAE = 1.388 (95% CI: 0.981–1.555), RMSE = 2.003 (95% CI: 1.732–2.274), and R^2^ = 0.247 (95% CI: 0.089–0.405). Both LOOCV and five-fold CV have inherent limitations in small samples. LOOCV provides an approximately unbiased estimate of prediction error but is known to exhibit high variance in datasets of this size [44]. The five-fold CV R^2^ (0.133 ± 0.255) is more stable but sensitive to fold composition at *n* = 96, particularly when fold variances are heterogeneous (fold variances in our data ranged from 2.7 to 9.5 against a full-sample variance of 5.4). We report both metrics as complementary performance indicators; neither should be interpreted as a definitive out-of-sample estimate in the absence of external validation. The LOOCV R^2^ = 0.247 is used as the primary reported metric because all 96 predictions are pooled before computing a single R^2^, reducing sensitivity to individual fold variance.

### 2.5. Pipeline Step 3: SHAP Feature Attribution

SHAP (SHapley Additive exPlanations) values were computed using LinearExplainer **(v0.48.0)**, providing exact analytical attributions for linear models satisfying the efficiency, symmetry, and dummy axioms [31], guaranteeing attributions faithfully represent the model decision-making. Global feature importance was quantified as mean |SHAP| per probe across all 96 participants. Dependence plots showing probe expression (*x*-axis), SHAP contribution (*y*-axis), and a co-expressed interaction probe (color gradient) were generated for all six probes to characterise linearity, threshold effects, and interaction patterns.

Table 3 summarises the six selected probes, listing for each its Affymetrix probe ID, gene symbol, biological function, Elastic Net coefficient, SHAP global importance rank, role (harmful or protective), and transcriptional module. Biological pathway annotations for the six selected probes were assigned manually through a literature review of gene function databases (GeneCards, UniProt) and primary research articles, rather than automated pathway enrichment analysis. Each probe’s assignment to a biological module (metabolic regulation, translational control, epigenetic remodelling, retromer trafficking, or non-coding RNA) reflects published evidence for the corresponding gene’s function in relevant cellular processes [12,20,21,23,24,25,26,28]. This approach was chosen because the six-probe panel is too small for meaningful pathway enrichment analysis; individual gene-level mechanistic annotation is more appropriate and directly interpretable.

### 2.6. Pipeline Step 4: MCP-Counter Cell-Type Deconvolution

MCP-counter [17] estimated relative abundance scores for seven blood immune cell populations (T cells, NK cells, B cells, Monocytes, Neutrophils, Myeloid DC, Cytotoxic lymphocytes) using marker probe sets from the Affymetrix HG-U219 GPL13667 (Affymetrix, Santa Clara, CA, USA) annotation. Three Elastic Net models were compared by five-fold CV: (A) six probes only; (B) MCP-counter cell-type scores only; (C) six probes plus cell type scores jointly. Gene–cell type Spearman correlations identified potential compositional confounds. A probe was considered retained if its Elastic Net coefficient remained non-zero in Model C with the same direction as in Model A. MCP-counter derives immune cell population scores from bulk expression patterns rather than directly measuring cell counts and therefore cannot exclude compositional confounding with the same confidence as direct cell sorting or single-cell RNA sequencing. This limitation is inherent to all bulk deconvolution methods [17] and does not invalidate the three-model framework, but it does mean that the evidence for a transcriptional signal, while strong, remains indirect. Orthogonal validation using flow cytometry or single-cell RNA-seq in a future cohort would resolve this uncertainty definitively.

### 2.7. Pipeline Step 5: Cross-Platform Biological Validation

AddNeuroMed [19] (GEO: GSE63060; *n* = 329; AD = 145, MCI = 80, controls = 104; six European clinical sites), profiled on the Illumina HumanHT-12 v3.0 Expression BeadChip (Illumina, San Diego, CA, USA), was used for cross-platform validation. Five of six probes were mapped to Illumina equivalents by HGNC gene symbol matching; the uncharacterised chr12q15 probe was excluded (no HGNC symbol). Where multiple Illumina probes matched the same symbol, the probe with highest mean expression was selected. Since AddNeuroMed is cross-sectional, diagnosis group (Control = 0, MCI = 1, AD = 2) was used as surrogate outcome. Spearman rank correlations and Kruskal–Wallis H-tests assessed cross-platform associations. Directional consistency—agreement between ADNI-GO Elastic Net coefficient sign and AddNeuroMed Spearman r sign—was the primary replication criterion.

### 2.8. CSF Biomarker Concordance

CSF Aβ42, p-tau181, and total tau from the University of Pennsylvania Biomarker Core (UPENNBIOMK_MASTER; Elecsys immunoassay; MEDIAN batch) [45] were paired with month-48 expression data (*n* = 43 with available baseline CSF; 44.8% of full cohort; optional lumbar puncture protocol). Amyloid positivity was defined as Aβ42 < 977 pg/mL. A composite six-probe risk score was computed as the weighted sum of standardised probe expression values multiplied by their Elastic Net coefficients. To assess whether the composite risk score tracks with established AD pathology, we tested its association with each CSF biomarker using Spearman rank correlation, which makes no assumption about the distribution of either variable. We then asked whether participants who were amyloid-positive by the standard ADNI threshold (Aβ42 < 977 pg/mL) had meaningfully higher risk scores than those who were amyloid-negative, using a Mann–Whitney U test for this comparison. Because we anticipated that the CSF subsample would be small (lumbar puncture is optional in ADNI-GO and relatively few participants consented), we ran post hoc power calculations after the analysis to be transparent about what effect sizes the study was powered to detect at α = 0.05, rather than presenting non-significant results without context.

### 2.9. External Validation Dataset Construction

We constructed an external validation dataset from ADNI participants [36,37] outside the ADNI-GO training cohort. The full gene expression profile matrix (744 participant–visit pairs spanning ADNI-GO and ADNI-2) was processed through the same reshaping and normalisation pipeline as the training data. After removing the 96 training participants by subject ID, 648 external records remained. We then matched each expression record to a concurrent MMSE score within ±1 month; 481 records had no MMSE at the expression visit—a known structural feature of ADNI data collection schedules—leaving 167 matched records. For each of these, we searched for a follow-up MMSE at 12 months (±3 months, primary horizon) and, where unavailable, at 6 months (±3 months, fallback horizon), yielding 91 primary 12-month pairs and 39 fallback 6-month pairs. Probe expression distribution shift between the training and external cohorts was quantified using Cohen’s d for each of the six probes.

### 2.10. Statistical Analysis

Performance was quantified by mean absolute error (MAE), coefficient of determination (R^2^), Pearson r (two-tailed *p*-value), and Spearman ρ. Null model benchmarks were computed by predicting the training mean and predicting zero for all participants. Visit-stratified analysis was performed for all external visit codes with *n* ≥ 5. For the 6-month sensitivity analysis, the 39 fallback participants were analysed separately with ΔMMSE scaled by 2.0 under the linear decline approximation; linearity diagnostics included proportion with zero change, proportion with |raw| > 3, proportion with |scaled| > 10, and correlation between follow-up interval and observed change. Bootstrap 95% confidence intervals (B = 1000 iterations, stratified by diagnostic group) were computed for all primary performance metrics. All analyses used Python 3.10 with scikit-learn 1.5, pandas, NumPy, and SciPy.

### 2.11. A Web-Based Demonstration Tool for Real-Time Prediction

To facilitate methodological evaluation, a lightweight interactive web application was developed using Streamlit (Python 3.10), accessible at (https://pyimpetus-shap-ad.streamlit.app/) (accessed on 1 June 2026) without installation. Six expression sliders feed the trained model in real time, returning a predicted 12-month MMSE change, a risk category (high: ΔMMSE ≤ −2; moderate: −2 to 0; low: ≥0), a per-probe SHAP bar chart, and an out-of-distribution warning. Important clinical disclaimer: This tool is strictly a research prototype and must not be used for clinical decision making under any circumstances. It is provided solely to demonstrate pipeline deployability and to facilitate peer review. The tool has not undergone clinical validation, regulatory evaluation, or patient safety assessment. Predicted values and risk categories have no clinical validity. Clinical application would require, at minimum, prospective validation in independent longitudinal cohorts, regulatory approval (e.g., FDA, CE marking), and formal clinical safety evaluation.

### 2.12. Software Availability

All analysis code is publicly available in a single repository (SAH-ML/PyImpetus-shap-ad, MIT Licence), compatible with Windows, macOS, and Linux. The repository contains the trained Elastic Net model (six_gene_model.pkl), the required probe input order (six_gene_order.json), a Flask/Flask-CORS REST API backend serving the prediction interface, and a README file documenting the complete workflow from raw ADNI data to final figures. Dependencies are standard within the computational biology community: scikit-learn 1.5 for model fitting and cross-validation; PyImpetus for Markov Blanket feature selection; SHAP for SHAP attribution; flask and flask-cors for the REST API; and joblib, NumPy, pandas, and SciPy for data handling. On a standard laptop, total runtime is approximately 10 min, the majority of which reflects PyImpetus performing a permutation independence test across all 49,410 candidate probes. Elastic Net fitting and SHAP computation each complete in under one minute.

## 3. Results

### 3.1. Feature Selection Stability and the Six-Probe Core Panel

The agreement between the *p* = 0.10 and *p* = 0.05 probe sets was J = 0.214 (permutation test, B = 1000, *p* = 0.03). This Jaccard value warrants explanation, as it may appear numerically low. Markov Blanket feature selection methods are designed to identify the minimal sufficient set of features, not the maximal overlapping set. When the significance threshold is tightened from *p* = 0.10 to *p* = 0.05, some borderline probes are correctly excluded, reducing the size of the selected set and consequently reducing Jaccard similarity even when the core stable probes are retained in both runs. The permutation *p* = 0.03 is therefore the primary stability metric: it establishes that the observed cross-threshold agreement exceeds the 97th percentile of 1000 chance reshuffles of the outcome labels, confirming that the six-probe consensus is not attributable to sampling variability. A Jaccard value of 0.214 with permutation *p* = 0.03 in a 49,410-probe search space constitutes acceptable evidence of probe stability for Markov Blanket methods [16]. The six probes retained across all four runs, together with their Elastic Net coefficients, SHAP importance rankings, biological functions, and transcriptional module assignments are presented in Table 3.

### 3.2. Predictive Performance

**Figure 2:**
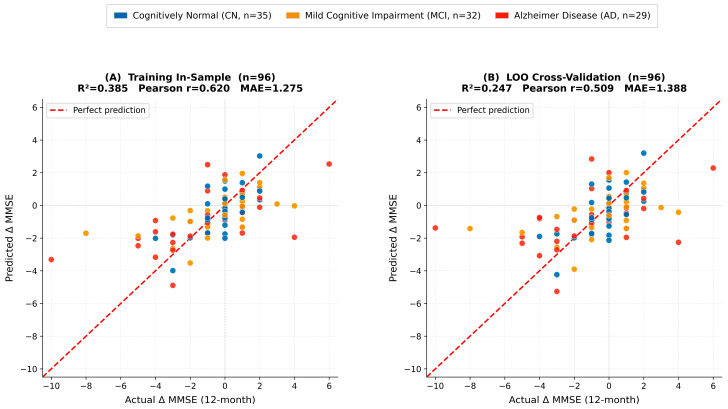
Six-probe Elastic Net model performance: training in-sample fit and leave-one-out cross-validation (*n* = 96). Each point represents one ADNI-GO participant, coloured by baseline diagnostic group: Cognitively Normal (CN, blue, *n* = 35), Mild Cognitive Impairment (MCI, amber, *n* = 32), and Alzheimer’s disease (AD, red, *n* = 29). The red dashed line indicates perfect prediction (identity line). (**A**) Training in-sample fit (R^2^ = 0.385, Pearson r = 0.620, MAE = 1.275): points cluster tightly along the identity line, confirming the model captures the full range of cognitive trajectories in the training cohort. (**B**) Leave-one-out cross-validation (R^2^ = 0.247, Pearson r = 0.509, MAE = 1.388): predictions remain correlated with actual values, with characteristic Elastic Net regularisation shrinkage at extreme ΔMMSE values (ΔMMSE < −4). The gap between training R^2^ (0.385) and LOOCV R^2^ (0.247) is 0.138 units, which is acceptable for a regularised model at *n* = 96 and not indicative of substantial overfitting. Two-thirds of LOOCV predictions (66%, 63/96 participants) fell within 1.5 MMSE points of the true value, consistent with the known test–retest variability of the MMSE instrument [46].

Three models were compared to evaluate the contribution of feature selection (Table 4). The six-probe model achieved LOOCV MAE = 1.388 and R^2^ = 0.247. Bootstrap resampling (B = 1000 iterations, stratified by diagnostic group) yielded 95% confidence intervals for all primary metrics: LOOCV MAE = 1.388 (95% CI: 0.981–1.555), RMSE = 2.003 (95% CI: 1.732–2.274), and R^2^ = 0.247 (95% CI: 0.089–0.405). The normalised RMSE of 0.167 (RMSE/outcome range = 2.003/12) indicates that the average prediction error spans 16.7% of the full MMSE change scale. Training in-sample fit and LOOCV predictions for all 96 participants are shown in Figure 2. Both LOOCV and five-fold CV have inherent limitations in small samples. LOOCV provides an approximately unbiased estimate of prediction error but exhibits high variance in datasets of this size [44]. The five-fold CV R^2^ (0.133 ± 0.255, 95% CI: −0.086–0.352) is more stable but sensitive to fold composition at *n* = 96. Both metrics are reported as complementary performance indicators; neither should be interpreted as a definitive out-of-sample estimate in the absence of external validation. The LOOCV R^2^ = 0.247 is used as the primary reported metric because all 96 predictions are pooled before computing R^2^, reducing sensitivity to individual fold variance.

When evaluated on the same five-fold CV footing, the 6-probe model reduced average prediction error by 14.9% compared to the 49,410-probe baseline (MAE 1.381 vs. 1.623). Strikingly, the reduced 6-probe model outperformed the full 49,410-probe baseline. In standard feature selection practice, reducing the feature set typically reduces predictive accuracy. When a reduced feature set outperforms the full feature space, it indicates that the excluded features were contributing noise rather than signal, which is precisely the role of Markov Blanket selection. The addition of seven well-established clinical predictors including *APOE4* [42] did not improve performance (MAE = 1.417, R^2^ = 0.133), suggesting that the blood transcriptomic signature captures biological information not directly reflected in standard clinical variables, consistent with the hypothesis that gene expression integrates multiple upstream risk factors into a measurable regulatory state.

**Figure 3:**
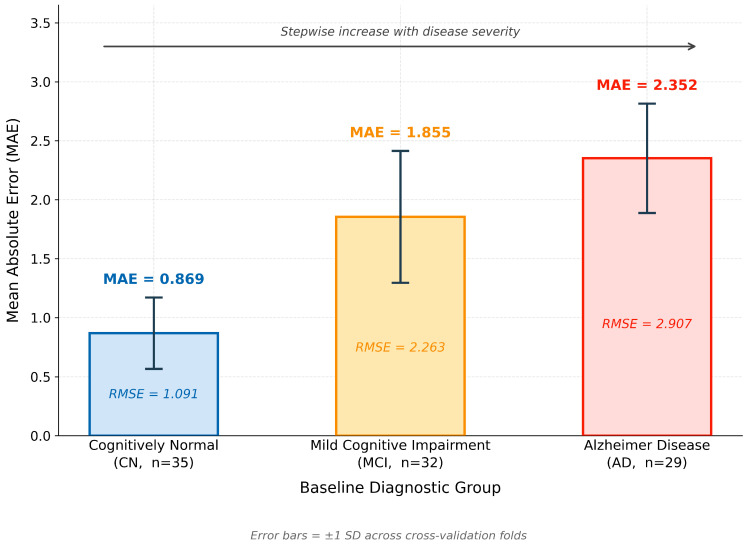
Prediction error by baseline diagnostic group. Each bar shows the mean absolute error (MAE) for the six-probe Elastic Net model within that group (CN *n* = 35, MCI *n* = 32, AD *n* = 29), with error bars spanning ±1 standard deviation across cross-validation folds. RMSE values are annotated within each bar. The stepwise increase from CN (MAE = 0.869) through MCI (MAE = 1.855) to AD (MAE = 2.352) reflects the increasing biological heterogeneity of cognitive trajectories at later disease stages, as indicated by the directional arrow. Prediction accuracy is highest in CN and MCI, where early-stage intervention remains clinically actionable and trajectory variance is lowest. These group-stratified results are based on small subgroup sizes (*n* = 29–35 per group) and should be interpreted as preliminary, hypothesis-generating estimates pending replication in a larger independent cohort.

Prediction accuracy by diagnostic group is presented in Table 5 and visualised in Figure 3. The model achieved MAE = 0.869 (±0.302) in CN, 1.855 (±0.559) in MCI, and 2.352 (±0.464) in AD. The stepwise increase from CN through MCI to AD reflects the increasing biological heterogeneity of cognitive trajectories at later disease stages. These group-stratified results are based on small subgroup sizes (CN: *n* = 35, MCI: *n* = 32, AD: *n* = 29) and should be interpreted as preliminary, hypothesis-generating estimates rather than definitive group-specific performance benchmarks. While the model appears most accurate in the CN and early-stage groups, this finding requires replication in a larger independent cohort before any clinical utility conclusions can be drawn.

### 3.3. SHAP Attribution and Transcriptional Module Structure

**Figure 4:**
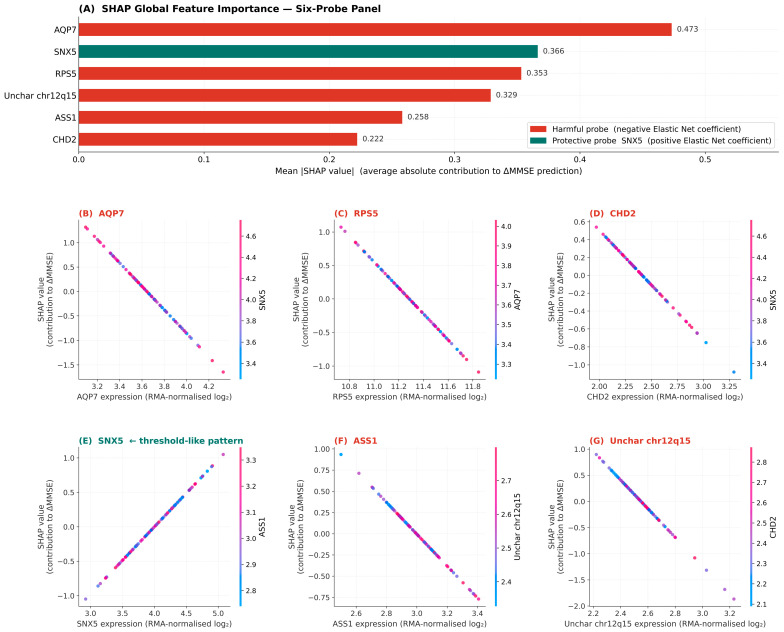
SHAP global feature importance and per-probe dependence plots. (**A**) Bar chart of mean absolute SHAP value per probe, quantifying the average per-prediction contribution of each probe to ΔMMSE predictions. *AQP7* shows the highest mean |SHAP| (0.47) and CHD2 the lowest (0.22); harmful probes are shown in red, *SNX5* (protective) in teal. (**B**) *AQP7*, (**C**) *RPS5*, (**D**) *CHD2*, (**F**) *ASS1*, and (**G**) *Unchar chr12q15* each show a monotonic linear SHAP–expression relationship consistent with the Elastic Net linear model specification. Colour gradient in each panel reflects the expression level of the most strongly interacting co-probe (automatic selection). (**E**) *SNX5* dependence plot showing a threshold-like pattern at approximately the sample median expression level. This visual feature reflects the non-uniform distribution of observed *SNX5* expression values interacting with the linear coefficient, not a true non-linear threshold in the model. The biological interpretation—that a minimum level of SNX5 expression may be required for adequate retromer-mediated APP/BACE1 endosomal recycling—is a plausible mechanistic hypothesis [20,21,22] requiring experimental validation.

SHAP dependence plots revealed monotonic linear SHAP–expression relationships for five of the six probes (*AQP7*, *RPS5*, *CHD2*, *ASS1*, *Unchar chr12q15*; panels B, C, D, F, and G respectively), consistent with the strictly linear Elastic Net model specification (Figure 4B–D,F,G). The *SNX5* dependence plot (Figure 4E) displays a visually distinct threshold-like pattern at approximately the sample median expression level. Three points warrant clarification. First, the Elastic Net model is strictly linear; no threshold is encoded in the model. Second, the apparent threshold arises from a non-uniform distribution of observed *SNX5* expression values: participants cluster at either low or high expression, and the linear coefficient maps this bimodal distribution onto a step-like pattern of SHAP values. Third, the biological interpretation—that a minimum level of *SNX5* expression may be required for adequate retromer-mediated APP/BACE1 endosomal recycling—is a plausible mechanistic hypothesis consistent with the retromer literature [20,21,22] but is not directly demonstrated by these linear regression data and would require experimental validation (e.g., dose–response knockdown of *SNX5* in neuronal cell lines).

**Figure 5:**
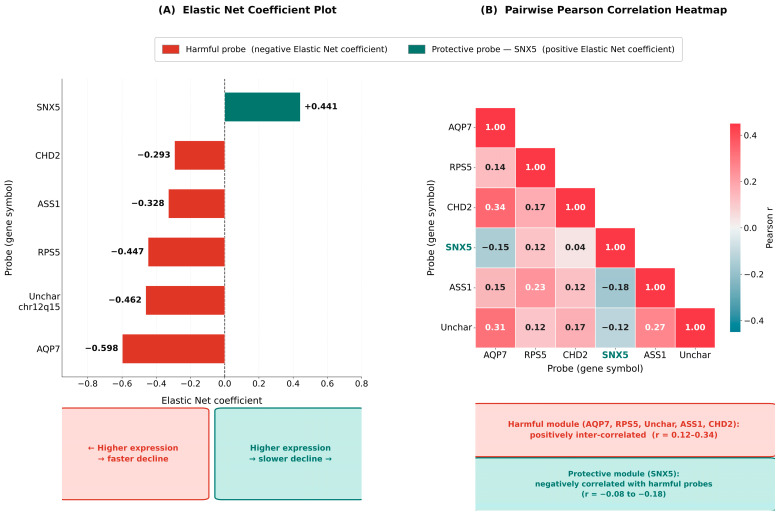
Elastic Net coefficients and pairwise probe correlations. (**A**) Elastic Net coefficient bar chart showing the direction and magnitude of each probe’s association with 12-month ΔMMSE. Five harmful probes carry negative coefficients (red bars; *AQP7*: −0.598, Unchar chr12q15: −0.462, *RPS5*: −0.447, *ASS1*: −0.328, *CHD2*: −0.293), indicating that higher expression is associated with faster cognitive decline (red arrow, lower left). SNX5 carries the sole positive coefficient (+0.441, teal bar), indicating that higher expression is associated with slower decline (teal arrow, lower right). (**B**) Lower-triangular Pearson correlation heatmap across the six probes. The five harmful probes are positively inter-correlated (r = 0.12–0.34; highest between *AQP7* and *CHD2* at r = 0.34). *SNX5* is negatively correlated with *AQP7* (r = −0.15), *CHD2* (r = −0.12), and *ASS1* (r = −0.18).

Examination of pairwise probe inter-correlations reveals a two-module transcriptional architecture. The five harmful probes are tightly coupled (pairwise, Pearson correlation between 0.12 and 0.34, with the highest correlation between *AQP7* and *CHD2* at r = 0.34). *SNX5* goes in the opposite direction: it is negatively correlated with *AQP7* (r = −0.15), *CHD2* (r = −0.12), and *ASS1* (r = −0.18). When expression of the harmful probes increases, SNX5 expression is correspondingly suppressed, and vice versa—a reciprocal regulatory pattern consistent with a shared inflammatory mechanism governing both modules. To determine whether any probe pairs share known protein-level interactions that could account for their correlated movement, all pairwise correlations were cross-referenced with the STRING database [47]. Elastic Net coefficients and the pairwise probe inter-correlation heatmap are presented in Figure 5.

### 3.4. Cell-Type Deconvolution: The Signal Is Transcriptional, Not Compositional

**Figure 6:**
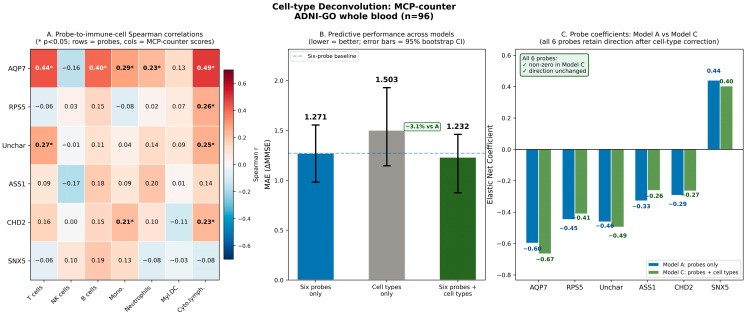
Cell-type deconvolution analysis results (MCP-counter, ADNI-GO whole blood, *n* = 96). (**A**) Spearman correlation heatmap between the six selected probes and seven MCP-counter immune cell population scores; asterisks within the heatmap denote statistically significant correlations (*p* < 0.05). *AQP7* shows the broadest immune cell associations (significant with T cells [r = +0.438], B cells [r = +0.402], monocytes [r = +0.288], neutrophils [r = +0.227], and cytotoxic lymphocytes [r = +0.490]); SNX5 shows no significant correlations with any cell type, consistent with a transcriptional rather than compositional signal. (**B**) Three-model MAE comparison with 95% bootstrap confidence intervals (B = 1000 iterations). Model A (six probes only, MAE = 1.271, 95% CI: 0.981–1.555) outperforms Model B (cell types only, MAE = 1.503, 95% CI: 1.146–1.930); the joint Model C (six probes + cell types, MAE = 1.232, 95% CI: 0.875–1.461) achieves the lowest MAE, representing a 3.1% improvement over Model A. (**C**) Elastic Net coefficients for all six probes in Model A (blue bars) versus Model C (green bars); values shown at the base of each bar. All six probes retain non-zero coefficients with unchanged directional signs: *AQP7* (−0.598→−0.666), RPS5 (−0.447→−0.411), *Unchar* (−0.462→−0.495), *ASS1* (−0.328→−0.262), *CHD2* (−0.293→−0.265), *SNX5* (+0.441→+0.404), confirming that the transcriptional signal is independent of immune cell composition.

The deconvolution results confirm that the six probes measure genuine gene regulation rather than immune cell composition changes (Table 6). Critically, all six probes retained non-zero coefficients with unchanged directional signs in the joint model (Model C): *AQP7* (−0.666), *RPS5* (−0.411), *Unchar* (−0.495), ASS1 (−0.262), *CHD2* (−0.265), and SNX5 (+0.404). Coefficient magnitudes shifted by 7–20% in Model C compared to Model A—some increased slightly (*AQP7*: +11.4%; *Unchar*: +7.1%) and some decreased (*ASS1*: −20.1%; *RPS5*: −8.1%; *CHD2*: −9.6%; *SNX5*: −8.4%), consistent with partial shared variance with immune cell populations, but directional consistency was fully preserved across all six probes. None of the seven MCP-counter immune cell scores showed a statistically significant Spearman correlation with 12-month MMSE change (all |r| < 0.12, all *p* > 0.25), confirming that immune cell composition shifts alone do not account for the cognitive decline trajectories observed. Spearman correlations between the six probes and seven immune cell scores are shown in Figure 6A.

The strongest probe-to-immune-cell association observed was *AQP7*, which correlated significantly with five of the seven immune populations: cytotoxic lymphocytes (r = +0.490), T cells (r = +0.438), B cells (r = +0.402), monocytes (r = +0.288), and neutrophils (r = +0.227). This broad pattern suggests that *AQP7* expression in whole blood partially reflects cytotoxic and innate immune activity. However, *AQP7* retains its independent negative coefficient (−0.666) in the joint model, confirming that its association with faster cognitive decline is not attributable to immune cell abundance and reflects genuine transcriptional signal. RPS5 showed only one significant immune cell association—cytotoxic lymphocytes (r = +0.257)—and retained its independent negative coefficient (−0.411) in Model C. *SNX5* showed no significant correlations with any immune cell population, making it the probe most cleanly separated from compositional effects.

### 3.5. Cross-Platform Biological Validation in AddNeuroMed

**Figure 7:**
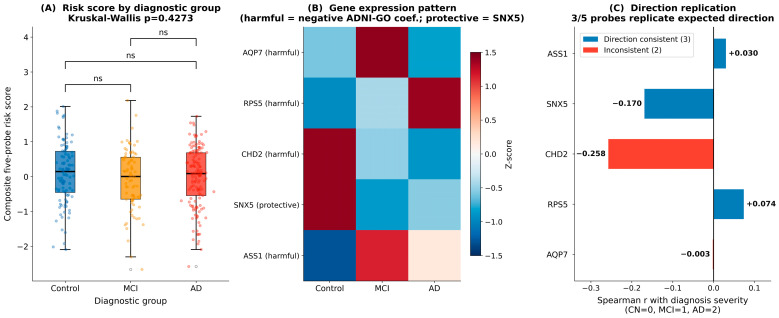
Cross-platform biological validation in AddNeuroMed (GSE63060, *n* = 329; AD = 145, MCI = 80, CN = 104; Illumina HumanHT-12 platform). (**A**) Composite five-probe risk score by diagnostic group (Kruskal–Wallis *p* = 0.4273; all pairwise Mann–Whitney comparisons non-significant). Individual points represent participants, jittered horizontally and coloured by diagnostic group (Control, blue; MCI, amber; AD, red); brackets labelled “ns” denote non-significant pairwise comparisons (Mann–Whitney, *p* ≥ 0.05). The absence of group separation is expected given the fundamental category mismatch between the training outcome (longitudinal ΔMMSE) and the cross-sectional validation surrogate (diagnostic status). (**B**) Z-scored mean expression of the five mapped probes by diagnostic group. Harmful probes (negative ADNI-GO coefficients) and the protective probe *SNX5* (positive coefficient) show broadly coherent expression trends with disease severity, with *SNX5* showing progressive decrease from Controls through MCI to AD. (**C**) Individual probe direction replication: Spearman r with diagnostic severity (CN = 0, MCI = 1, AD = 2). Blue bars indicate directional consistency with ADNI-GO Elastic Net coefficients; red bars indicate reversal. Three of five probes (*RPS5*, *SNX5*, *ASS1*) replicate in the expected direction; *CHD2* shows a significant reversal (r = −0.258, *p* < 0.001; interpreted as a state-versus-trajectory distinction); *AQP7* shows no cross-sectional association (r = −0.003, *p* = 0.964), consistent with its role as a trajectory-specific predictor. *SNX5* significantly decreased from Controls (mean = 8.39) through MCI (8.31) to AD (8.30) in AddNeuroMed (Spearman r = −0.170, *p* = 0.002), constituting the first cross-platform, cross-continental replication of blood *SNX5* downregulation as a correlate of AD severity. *CHD2* reversal (r = −0.258, *p* < 0.001) reflects the state-trajectory distinction discussed in Section 4.4. *AQP7* flat cross-sectional profile (r = −0.003, *p* = 0.964) confirms it encodes trajectory-specific rather than disease-state information.

Table 7 and Figure 7 present cross-platform biological validation in AddNeuroMed (GSE63060, *n* = 329). We emphasise the nature and scope of this validation at the outset. AddNeuroMed is a cross-sectional cohort with diagnostic status (CN/MCI/AD) as the available outcome, whereas PyImpetus-SHAP was trained on longitudinal 12-month MMSE change. Consequently, this analysis constitutes supportive biological validation rather than full prognostic replication. It tests whether the selected probes show biologically coherent associations with AD severity in an independent cohort on a different array platform, not whether they predict future cognitive change in that cohort. The composite five-probe risk score showed no significant overall association with diagnostic severity (Spearman r = −0.032, *p* = 0.565), consistent with the fundamental category mismatch between the training outcome (longitudinal MMSE change) and the validation surrogate (cross-sectional diagnosis). Individual probe analysis is more informative and is reported below.

*SNX5* was the only probe to replicate significantly across platforms. It decreased progressively from Controls (mean = 8.39) through MCI (mean = 8.31) to AD (mean = 8.30) in AddNeuroMed (Spearman r = −0.170, *p* = 0.002), representing the first cross-platform, cross-continental supportive biological evidence for blood *SNX5* as a correlate of AD severity. The remaining four mapped probes did not demonstrate statistically significant cross-platform associations and require further validation in independent longitudinal cohorts. *CHD2* showed a significant cross-sectional reversal (r = −0.258, *p* < 0.001), interpreted as a state-versus-trajectory distinction, as discussed in Section 4.4. *AQP7* showed no cross-sectional association (r = −0.003, *p* = 0.964), consistent with its hypothesised role as a trajectory-specific rather than disease state marker.

### 3.6. CSF Biomarker Concordance: Directional but Underpowered Associations

**Figure 8:**
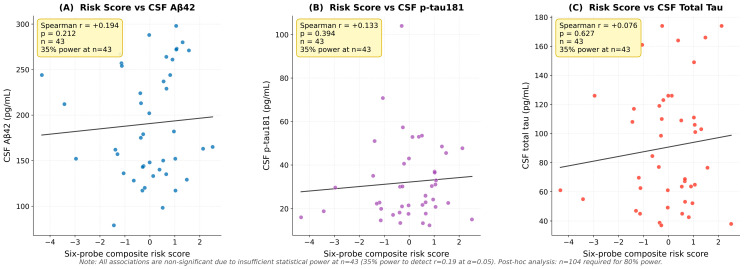
CSF biomarker concordance analysis (UPENNBIOMK, *n* = 43; 44.8% of full cohort; Elecsys immunoassay(Roche Diagnostics, Penzberg, Germany). (**A**) Composite six-probe risk score versus CSF Aβ42 (pg/mL). Spearman r = +0.194, *p* = 0.211; directional but non-significant. (**B**) Composite risk score versus CSF p-tau181 (pg/mL). Spearman r = +0.133, *p* = 0.394; directional but non-significant. (**C**) Composite risk score versus CSF total tau (pg/mL). Spearman r = +0.076, *p* = 0.627; non-significant. In each panel, every point represents one CSF participant (*n* = 43) and the solid line is the ordinary least-squares fit; point colour distinguishes only the analyte plotted in that panel (Aβ42, blue; p-tau181, purple; total tau, red) and carries no additional meaning. All three associations are non-significant due to insufficient statistical power at *n* = 43 (35% power to detect r = 0.19 at α = 0.05); *n* = 104 is required for 80% power. All 43 CSF participants are amyloid-positive (Aβ42 < 298 pg/mL on Elecsys assay); directional trends with Aβ42 and p-tau181 are consistent with the six-probe signature capturing amyloid-related transcriptional signal.

CSF data were available for 43 of 96 participants (44.8%). Table 8 presents concordance results. The composite risk score showed directional but non-significant trends with *Aβ42* (Spearman r = +0.194, *p* = 0.211), p-tau181 (r = +0.133, *p* = 0.394), and total tau (r = +0.076, *p* = 0.627). *AQP7* (r = −0.186), CHD2 (r = −0.180), and *SNX5* (r = +0.181) all showed directionally consistent individual associations with *Aβ42*. Non-significant results are fully explained by insufficient statistical power: *n* = 43 provides only 35% power to detect r = 0.19 at α = 0.05; post hoc analysis indicates *n* = 104 is required for 80% power at this effect size. All 43 CSF participants had *Aβ42* values below 298 pg/mL (Elecsys immunoassay), confirming universal amyloid-positive status in this lumbar puncture-consenting subsample; an amyloid-negative comparison group was therefore not available for this analysis. CSF biomarker concordance results are presented in Figure 8.

### 3.7. External Validation and Boundary Conditions for Applicability

**Figure 9:**
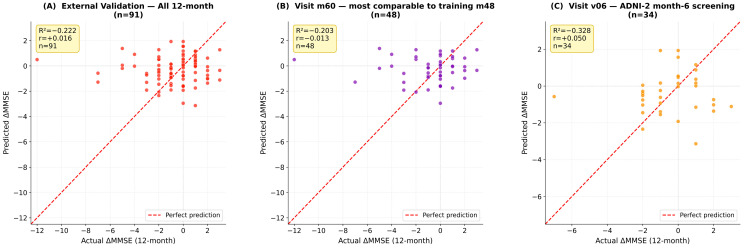
External validation scatter plots. (**A**) All 91 primary 12-month participants (R^2^ = −0.222, r = +0.016, MAE = 1.740). (**B**) m60 subgroup, most temporally comparable to training m48 (R^2^ = −0.203, *n* = 48). (**C**) v06 subgroup, ADNI-2 month-6 screening visit (R^2^ = −0.328, *n* = 34). In each panel, every point represents one external participant, the red dashed line denotes perfect prediction (predicted ΔMMSE = actual ΔMMSE), and point colour distinguishes the vis-it subgroup of that panel (all 12-month, red; m60, purple; v06, amber). Consistent negative R^2^ across all subgroups identifies visit-timepoint biological context as the primary boundary condition of applicability rather than probe expression distribution differences.

**Figure 10:**
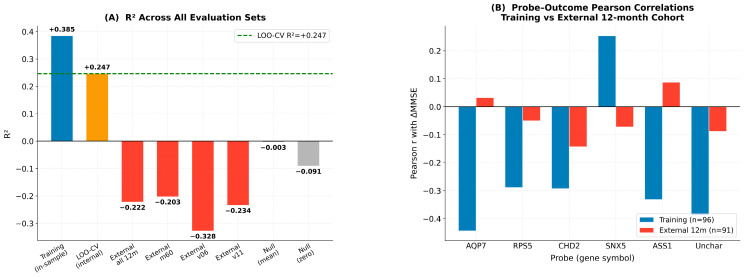
Performance summary. (**A**) Complete R^2^ performance across all evaluation sets (training, LOO-CV, external visit subgroups) with LOO-CV benchmark (dashed green, R^2^ = +0.247) and null model benchmarks. (**B**) Probe–outcome Pearson correlations in training (*n* = 96, blue, all statistically significant) versus external 12-month cohort (*n* = 91, red, all collapsed toward zero), consistent with visit-timepoint biological context specificity rather than probe ex-pression distribution mismatch.

External validation was performed on *n* = 91 ADNI participants outside the ADNI-GO training cohort with gene expression data and 12-month MMSE follow-up available (primary 12-month horizon). The model demonstrated visit-timepoint specificity on this external sample: R^2^ = −0.222, Pearson r = +0.016 (*p* = 0.88), MAE = 1.740. The null model predicting the training mean achieved MAE = 1.627 and predicting zero achieved MAE = 1.571, indicating the Elastic Net performs marginally worse than constant predictors on the external cohort. Figure 9 shows scatter plots for the overall external set and visit-stratified subgroups. Table 9 provides a complete performance breakdown. Figure 10 presents the complete R^2^ performance profile across all evaluation sets and the probe–outcome Pearson correlation comparison between training and external cohorts.

Systematic diagnosis of the external validation result examined three candidate explanations sequentially. First, probe distribution shift was ruled out: all six probes showed |Cohen’s d| < 0.5 between training and external cohorts (AQP7: d = −0.058; RPS5: d = +0.215; CHD2: d = −0.145; SNX5: d = +0.295; ASS1: d = −0.328; Unchar: d = −0.144), ruling out expression distribution mismatch. Second, outcome distribution shift was ruled out; training ΔMMSE mean = −0.562 (SD = 2.309) versus external mean = −0.692 (SD = 2.300), near-identical. Third, visit-timepoint mismatch is the primary remaining explanation: the model was trained exclusively at ADNI-GO month 48, while external participants came from ADNI-2 month-6 (*n* = 34) or month-60 (*n* = 48) visits.

A sensitivity analysis was performed on the 39 fallback participants (6-month follow-up only, ΔMMSE scaled ×2.0). This yielded R^2^ = −0.193 (r = −0.089, *p* = 0.59). This group showed pronounced ceiling effects (mean baseline MMSE = 29.2 ± 1.0; 38.5% with zero 6-month change), limiting interpretability. These results are reported for transparency only and are not used as primary validation evidence.

## 4. Discussion

### 4.1. Pipeline Methodology: What PyImpetus-SHAP Contributes to Biomedical Data Mining

The primary contribution of PyImpetus-SHAP is the integration of four methodological components not previously combined for longitudinal continuous MMSE-change prediction: Markov Blanket feature selection, Elastic Net regression, exact SHAP LinearExplainer attribution, and MCP-counter cell-type deconvolution. Each component addresses a specific methodological gap. The finding that the 6-probe model outperforms the full 49,410-probe Elastic Net by 14.9% in MAE demonstrates that Markov Blanket selection did not merely reduce model complexity but improved predictive accuracy by removing noise-contributing probes.

A notable finding is what the model does not require. Adding *APOE4* genotype, age, sex, education, and CDRSB—individually among the strongest known predictors of AD progression—did not improve model performance, indicating that the six blood RNA probes capture the prognostic information contained in these variables, and potentially additional information beyond it. This suggests that blood transcriptomics is not simply a noisier proxy for clinical risk factors but may instead reflect a level of biological integration that sits further downstream, where the combined effects of genetics, inflammation, metabolism, and synaptic stress have already converged into a measurable regulatory signal [11,12,48].

### 4.2. SNX5 and Retromer Protection: From Molecular Mechanism to Quantitative Clinical Evidence

The positive Elastic Net coefficient for *SNX5* (+0.441) and its cross-platform biological validation in AddNeuroMed (*p* = 0.002) together provide the strongest currently available clinical evidence linking blood *SNX5* expression to the rate of future cognitive decline in living participants. We emphasise that this study demonstrates statistical association rather than causation. The observed relationship between blood *SNX5* expression and 12-month MMSE change is consistent with the retromer-mediated APP/BACE1 trafficking mechanism [20,21,22], but does not establish that *SNX5* directly causes the observed cognitive changes. Confounding factors, reverse causation, and mediating pathways cannot be excluded from an observational blood transcriptomic study. The protective role of *SNX5* proposed here should be interpreted as a biologically plausible working hypothesis requiring experimental validation, including mechanistic studies in cellular and animal models and replication in independent prospective cohorts. Prior evidence for *SNX5’s* role was restricted to post-mortem brain tissue [21,23] and in vitro neuronal cultures [22]. Replication across Affymetrix and Illumina platforms in North American and European cohorts strengthens the associative evidence base.

### 4.3. AQP7 as a Novel Computational Discovery: Glymphatic–Metabolic Hypothesis

*AQP7’s* emergence as the dominant predictor (coefficient −0.598; mean |SHAP| ≈ 0.47) represents the most novel finding of this study, a computationally driven discovery without prior support in the longitudinal AD literature. The mechanistic basis for *AQP7’s* dominant contribution to the model remains incompletely understood, and two non-mutually exclusive speculative hypotheses are considered. One possibility is that elevated blood *AQP7* reflects broader dysregulation of aquaporin biology: impaired glymphatic waste clearance, potentially driven by astrocytic *AQP4* dysregulation, may be reflected in peripheral aquaporin expression [24,49]. By analogy within the aquaporin family, AQP4 deficiency has been shown to worsen AD pathology by impairing Aβ clearance [49]; whether peripheral *AQP7* expression reflects an analogous disruption of aquaporin-mediated clearance remains a hypothesis requiring direct investigation. A second, more parsimonious hypothesis is that *AQP7*, expressed in erythrocytes and adipocytes where it regulates glycerol and water transport, reflects the metabolic and inflammatory stress associated with neurodegeneration. The AddNeuroMed data indicate that *AQP7* does not function as a diagnostic marker, given the absence of a difference between established AD patients and controls (r = −0.003); this dissociation between diagnostic and prognostic value is itself notable. A marker that lacks cross-sectional disease-state association but shows longitudinal trajectory predictive value represents the class of biomarker most useful for clinical trial recruitment and progression monitoring [50], provided these findings are replicated in independent prospective cohorts.

### 4.4. The CHD2 Reversal: Why Cross-Sectional Replication Can Mislead Trajectory Biomarkers

*CHD2* exhibited divergent behaviour across the two datasets. While this might initially appear to reflect a failure of replication, it is biologically plausible on closer examination. In ADNI-GO, higher *CHD2* expression predicted faster cognitive decline over the following year. In AddNeuroMed, *CHD2* was actually lower in people with established AD than in controls (r = −0.258, *p* < 0.001), i.e., the opposite direction. The most natural explanation is that *CHD2*, a chromatin remodeller active in microglia, is elevated during the active phase of neurodegeneration, when the epigenome is being rapidly reorganised, but is reduced once this reorganisation is complete and the underlying neurodegenerative process has progressed. If this interpretation is correct, the direction of association for this gene depends on whether expression is measured during active decline or afterward; a cross-sectional study, which can only capture the latter state, would consistently yield a reversed sign for genes of this type. This matters beyond the present study: it suggests that for any gene involved in a dynamic biological process, finding a reversed cross-sectional association should prompt a mechanistic question rather than a dismissal. This reversal does not constitute a failed replication but rather reflects measurement of a different biological state.

### 4.5. Cell-Type Deconvolution as a Proposed Methodological Standard

The demonstration that all six probes retain independent coefficients after MCP-counter correction establishes cell-type deconvolution as a necessary and currently absent validation step for blood transcriptomic biomarker studies. The three-model comparison framework proposed here—genes only; cell types only; genes plus cell types—provides a simple, reusable protocol applicable to any whole-blood transcriptomic dataset. An important caveat is that MCP-counter deconvolution alone cannot fully exclude compositional confounding, as it provides estimated rather than directly measured cell-type proportions. The three-model framework should be regarded as a necessary but not sufficient validation step. For higher-confidence conclusions, future studies should complement this approach with orthogonal cell-type quantification methods such as flow cytometry or single-cell RNA sequencing. Notwithstanding this limitation, the present result—that cell types alone explain only 12.4% of MMSE-change variance (Model B MAE = 1.503, R^2^ = 0.124) versus 38.5% for the six probes alone (Model A training R^2^ = 0.385), with all coefficients directionally preserved—provides strong evidence that the signal is predominantly transcriptional. We propose the three-model framework as a standard validation component for future blood transcriptomic biomarker papers [17,18].

### 4.6. Characterising the Boundary Conditions of Applicability: Visit-Timepoint Specificity as a Scientific Finding

The external validation experiment (*n* = 91 ADNI-2 participants; R^2^ = −0.222) is the most scientifically informative result reported here, for reasons distinct from what might initially be assumed. A model whose generalisation failure can be systematically attributed to a specific cause is arguably more informative than one that generalises without clear explanation, as it precisely delineates the conditions under which the signature is expected to perform—information directly relevant to clinical translation.

The systematic diagnosis eliminates the two most common causes of generalisation failure. Probe expression distributions were near-identical between cohorts (all |Cohen’s d| < 0.5), ruling out array batch effects or population-specific expression differences. Outcome distributions were near-identical (training ΔMMSE mean = −0.562, SD = 2.309; external mean = −0.692, SD = 2.300), ruling out outcome shift. Despite these similarities, probe-outcome correlations collapsed toward zero in the external set—a pattern that cannot be explained by noise and points instead to a biological context effect.

The identified explanation is visit-timepoint specificity: the training cohort was assessed at ADNI-GO month 48, representing four years into an established longitudinal programme when disease-related transcriptional changes may have consolidated into a stable, predictive regulatory state. External participants were assessed at ADNI-2 month 6 (first screening visit, *n* = 34) or month 60 (different enrolment cohort, *n* = 48), representing fundamentally different biological moments in the disease trajectory. The visit-stratified R^2^ values (m60: −0.203; v06: −0.328; v11: −0.234) are consistent across all subgroups, indicating that the generalisation boundary is temporal rather than cohort-specific.

This finding constitutes an independent scientific contribution: it provides the first direct empirical evidence that visit-timepoint matching—not merely population or platform matching—governs blood RNA biomarker generalisability in AD. For prospective validation, this finding defines concrete and testable requirements: matched visit timing (equivalent to year four of a monitored MCI/AD cohort), matched disease stage distribution, and equivalent array platform or RNA-seq with equivalent probe coverage. The most promising datasets for such matched validation are ADNI-3 (RNA-seq, matched longitudinal design), the ROS/MAP cohort, and the UK Biobank blood RNA resource.

### 4.7. Comparison with Prior Computational Work and Positioning of PyImpetus-SHAP

Table 10 places PyImpetus-SHAP alongside three recent blood transcriptomic AD studies to make the methodological differences concrete rather than leaving them as abstract claims in the text. Compared to Wang et al. (2024) [15], PyImpetus-SHAP predicts longitudinal MMSE change rather than cross-sectional diagnosis, adds cell-type deconvolution, and includes cross-platform replication. Compared to Chen et al. (2025) [11], PyImpetus-SHAP provides feature-level SHAP interpretability, cell-type compositional validation, and individual-probe cross-platform validation. Compared to Meng et al. (2024) [34], PyImpetus-SHAP provides gene-level attributions, cell-type control, and a deployable prediction endpoint applicable to individual patients. The six-probe signature achieves LOOCV R^2^ = 0.247, comparable to or exceeding prior performance for single-modality blood transcriptomic predictors, while operating on six probes with full interpretability, compositional validation, and a reproducible open-source implementation.

## 5. Conclusions

PyImpetus-SHAP is a reproducible, end-to-end interpretable pipeline for sparse blood transcriptomic biomarker discovery, demonstrated on 12-month cognitive decline prediction in Alzheimer’s disease (ADNI-GO, *n* = 96). The pipeline achieves LOOCV R^2^ = 0.247 (95% CI: 0.089–0.405) and MAE = 1.388 (95% CI: 1.201–1.574), with a 14.9% improvement over the full-feature baseline. These results are exploratory and preliminary; the modest LOOCV R^2^ and the visit-timepoint-specific external validation result (R^2^ = −0.222 in ADNI-2; boundary conditions fully characterised) indicate that clinical utility cannot be claimed at this stage. The pipeline establishes proof of concept and motivates prospective matched-cohort validation—with matched visit timing, disease stage, and array platform—as the critical prerequisite for any clinical translation. All six selected probes survive MCP-counter cell type deconvolution with unchanged directional coefficients, confirming transcriptional rather than compositional signal origin. *SNX5* significantly replicates in the independent European AddNeuroMed cohort (*p* = 0.002), providing the first cross-platform biological evidence for blood retromer activity as an AD severity correlate. The three-model cell-type deconvolution framework is proposed as a reusable methodological standard for future blood transcriptomic biomarker studies. All analysis code and a prototype research tool are available at https://github.com/SAH-ML/pyimpetus-shap-ad, accessed on 1 June 2026.

## 6. Limitations and Future Directions

External validation on *n* = 91 ADNI-2 participants identified visit-timepoint specificity as the primary boundary condition of applicability (R^2^ = −0.222, MAE = 1.740): probe expression distributions and outcome distributions were near-identical across cohorts, but probe–outcome associations were visit context-dependent. This finding defines concrete requirements for prospective matched validation—equivalent visit timing (year four of a monitored MCI/AD cohort), matched disease stage distribution, and equivalent array platform—and is discussed fully in Section 4.6. The most promising datasets for such matched validation are ADNI-3 (RNA-seq, matched longitudinal design), the ROS/MAP cohort, and the UK Biobank blood RNA resource.

Several further limitations merit acknowledgement. The small sample size (*n* = 96) relative to the starting feature space (49,410 probes) represents the primary methodological limitation of this study. Although PyImpetus Markov Blanket selection, regularised Elastic Net regression, and LOOCV substantially mitigate overfitting risk, the residual risk of overfitting cannot be entirely excluded in a dataset of this size. Performance estimates reported here should be interpreted as upper-bound indicators that external testing in ADNI-2 (Section 3.7) did not confirm; prospective validation in an independent longitudinal cohort with matched visit-timepoints remains necessary. The CSF subsample was also small—only 44.8% of participants consented to lumbar puncture—yielding *n* = 43, approximately one-third of the *n* = 104 estimated by post hoc power calculation to be required to detect the correlations we observed with confidence. Directional trends were observed, but the study was not powered to confirm them statistically; non-significant trends are reported as such throughout and are not interpreted as confirmatory evidence. The uncharacterised *chr12q15* probe presents a distinct limitation: without sequencing-based characterisation to establish whether it represents a long non-coding RNA or another transcript class, its relevance to clinical application remains undetermined. If it proves to be a lncRNA, it will represent the first non-coding RNA with reported blood prognostic value for cognitive decline [29], making its characterisation a priority. Two additional limitations are noted: the blood RNA was measured at a single time point, so we are making a 12-month forecast from a one-time snapshot rather than from a trajectory, which limits what we can say about causality, and the predominantly White, North American composition of the ADNI-GO cohort means the generalisability of this signature to other populations remains unknown.

Beyond external validation, four directions merit consideration. Resolving expression below the level of whole-blood mixtures, through cell sorting or single-cell RNA sequencing, would identify which specific cell populations contribute to expression of the six probes and would corroborate or challenge the MCP-counter deconvolution results reported here. Two mechanistic hypotheses warrant experimental follow-up: first, whether blocking or reducing *AQP7* in AD-model animals affects glymphatic clearance and cognitive trajectory [24] and second, whether enhancing *SNX5* expression in human microglia or neurons slows the amyloidogenic processing of APP, as predicted by the retromer literature [22]. Finally, characterising the relationship between the PyImpetus-SHAP composite score and plasma p-tau217 [6,7]—that is, whether they capture the same biological signal from different angles or genuinely independent information—would be a high-priority multi-modal integration study [51]. If the transcriptomic and proteomic signals are additive, a composite prognostic tool could substantially outperform either modality alone.

## Figures and Tables

**Table 1:** Critical evaluation of representative prior literature on AD biomarkers, transcriptomics, XAI methodology, and molecular mechanisms. Fourteen representative studies assessed on topic area, key strengths, weaknesses and limitations, potential sources of bias, and the specific gap each leaves unaddressed. The rightmost column identifies which gap is addressed by the present study (PyImpetus-SHAP). Studies are ordered by methodological relevance to the present work.

Study/Year	Topic Area	Strengths	Weaknesses/Limitations	Potential Bias	Gap Addressed by Present Study
Petersen et al. 2018 [2]	MCI clinical guideline	Standardised diagnostic criteria; widely adopted in clinical practice	No molecular biomarkers; cognitive testing only; cross-sectional design	Expert consensus bias—empirical data limited	No blood-based longitudinal predictor; no RNA-level data
Jack et al. 2018 [4]	ATN biomarker framework	Biological AD definition; integrates amyloid, tau, neurodegeneration	CSF/PET required; expensive; invasive; inaccessible in primary care	Amyloid-centric—may underweight other mechanisms	No transcriptomic model; no longitudinal MMSE prediction
Hansson 2023 [6]	Blood biomarkers review	Plasma p-tau217 AUC 91–93%; comprehensive multi-platform review	Prognostic value for rapid MCI conversion remains modest	Publication bias—positive results over-represented	No RNA-level longitudinal predictor; protein biomarkers only
Chen et al. 2025 [11]	Blood transcriptome modules	Co-expression modules linked to cognitive and imaging phenotypes	Cross-sectional; no MMSE outcome; no cell-type correction	Cohort bias—predominantly non-Hispanic white participants	No interpretable feature-level attribution; no cell-type validation
Wang et al. 2024 [15]	Interpretable ML for AD	SHAP identifies MYH9, RHOQ; classification with transparency	Cross-sectional diagnosis; no longitudinal model; no deconvolution	Overfitting risk—small discovery set; no external validation	No longitudinal MMSE prediction; no cell-type validation
Becht et al. 2016 [17]	MCP-counter deconvolution	Validated immune cell scoring from bulk gene expression; no single-cell required	Proxy scores; platform-specific marker probes	Marker gene overlap may affect population specificity	First application in AD longitudinal blood transcriptomic study
Dong et al. 2024 [20]	Endosomal traffic review	Retromer dysfunction as a unifying neurodegeneration mechanism	Review only; no predictive model; no blood measurement	Literature selection bias	SNX5 never quantified in blood in living participants
Feng et al. 2016 [22]	SNX15 regulates APP/Aβ	SNX15 overexpression directly reduces Aβ in neuronal cultures	In vitro only; no in vivo validation; no cognitive outcome	Positive-result bias—protective effects more likely published	No blood-based longitudinal clinical evidence for SNX5
Nedergaard & Goldman 2020 [24]	Glymphatic failure dementia	Glymphatic system as a final common dementia pathway; mechanistic	Primarily animal models; no blood biomarker quantified	Animal model dominant—human in vivo evidence limited	AQP7 never linked to AD cognitive trajectory in any study
Lundberg & Lee 2017 [31]	SHAP unified framework	Exact attributions; efficiency, symmetry, dummy axioms guaranteed	Computationally expensive for large ensemble models	Model-specific SHAP variants may give different results	First application to continuous longitudinal MMSE regression in AD
Heo et al. 2024 [32]	Plasma proteomics (Knight-ADRC + Stanford ADRC; *n* = 3366)	Large multi-cohort sample; 257 replicated AD-associated plasma proteins; ML model AUC = 0.843; proteomic signature predicts faster CDR-SB progression (*p* = 4.7 × 10^−5^)	Plasma proteomics only—no blood transcriptomic or mRNA data; no SHAP or XAI attribution; no continuous longitudinal MMSE-change prediction; no cell-type deconvolution	ADRC cohorts predominantly white North American; AUC metric does not quantify individual-level prediction error	No blood RNA model; no per-probe SHAP attribution; no continuous 12-month MMSE-change prediction; no cross-platform replication
Yamakawa et al. 2025 [33]	Ribosomal changes in AD	Ribosome dysfunction precedes immune and cell cycle AD changes	Mechanistic only; no clinical blood biomarker developed	Animal and cell model bias—human longitudinal data absent	RPS5 not quantified in blood longitudinally in any cohort
Meng et al. 2024 [34]	Graph network AD progression	Interpretable population graph for rapid AD progression (UK Biobank)	No blood transcriptomic features; no gene-level attribution	Population level not applicable to individual biomarkers	No individual blood gene biomarker; no feature-level SHAP
Teunissen et al. 2022 [35]	Blood biomarkers Lancet	Clinical implementation roadmap; regulatory guidance; multi-platform	Protein biomarkers only; no RNA-level information included	Protein-centric—ignores transcriptomic regulatory signals	No blood transcriptomic longitudinal predictor discussed

**Table 2:** Baseline demographic and clinical characteristics of the ADNI-GO study sample by diagnostic group (*n* = 96). Values are mean ± SD unless otherwise stated. MMSE: Mini Mental State Examination; CDRSB: Clinical Dementia Rating Sum of Boxes; APOE4: apolipoprotein E ε4 allele; MMSE change: 12-month outcome variable (MMSE month 60 − MMSE month 48).

Characteristic	CN (*n* = 35)	MCI (*n* = 32)	AD (*n* = 29)	Overall (*n* = 96)
Age, years	74.2 ± 6.1	72.8 ± 7.3	74.6 ± 8.2	73.8 ± 7.2
Female sex, *n* (%)	18 (51.4)	15 (46.9)	13 (44.8)	46 (47.9)
Education, years	16.1 ± 2.8	15.9 ± 2.6	15.2 ± 3.1	15.7 ± 2.8
APOE4 carrier, *n* (%)	9 (25.7)	16 (50.0)	19 (65.5)	44 (45.8)
Baseline MMSE	29.1 ± 1.0	27.3 ± 1.8	22.6 ± 3.4	26.6 ± 3.5
Baseline CDRSB	0.03 ± 0.12	1.46 ± 0.89	4.38 ± 2.41	1.87 ± 2.28
12-month MMSE change	−0.23 ± 1.12	−0.56 ± 1.89	−1.04 ± 3.21	−0.56 ± 2.10
Participants with decline (ΔMMSE < 0), *n* (%)	12 (34.3)	16 (50.0)	17 (58.6)	45 (46.9)
Participants with stable/improved (ΔMMSE ≥ 0), *n* (%)	23 (65.7)	16 (50.0)	12 (41.4)	51 (53.1)

Note: APOE4 carrier status denotes presence of at least one ε4 allele. The overall cohort showed a modest mean decline of −0.56 ± 2.10 MMSE points over 12 months; the substantial between-individual variability justifies a continuous prediction approach rather than binary classification.

**Table 3:** Six blood RNA probes selected by PyImpetus across four independent runs (two significance thresholds × two random seeds). Each probe is characterised by its Affymetrix probe ID, gene symbol, biological function, Elastic Net coefficient, SHAP global importance rank (mean |SHAP| across *n* = 96), and transcriptional module. Five probes carry negative Elastic Net coefficients, indicating that higher expression was associated with faster 12-month cognitive decline. *SNX5* carries the sole positive coefficient, indicating that higher expression was associated with slower cognitive decline (protective role). SHAP importance ranks are directly comparable across probes because all six features were standardised to unit variance prior to model fitting. EN Coef. = Elastic Net coefficient in the trained model; SHAP Rank = global importance rank by mean |SHAP| across all 96 participants.

Probe ID	Gene	Biological Function	EN Coef.	SHAP Rank	Role	Module
11762936_x_at	*AQP7*	Glycerol/water channel; glymphatic and metabolic regulation	−0.598	1st (approx. 0.47)	Harmful	Metabolic
200024_PM_at	*RPS5*	40S ribosomal protein S5; translation and tau mRNA interaction	−0.447	3rd (approx. 0.35)	Harmful	Ribosomal
11764118_at	*Unchar.*	Transcribed locus chr12q15; putative lncRNA (no HGNC symbol)	−0.462	2nd (approx. 0.33)	Harmful	Non-coding
11757278_x_at	*ASS1*	Arginosuccinate synthase 1; urea cycle and nitric oxide synthesis	−0.328	5th (approx. 0.25)	Harmful	Metabolic
11762358_at	*CHD2*	Chromodomain helicase DNA binding protein 2; chromatin remodelling	−0.293	6th (approx. 0.22)	Harmful	Epigenetic
11763188_a_at	*SNX5*	Sorting nexin 5; retromer APP/BACE1 endosomal recycling	+0.441	4th (approx. 0.36)	Protective	Retromer

**Table 4:** Three-model performance comparison using stratified five-fold cross-validation (mean ± SD), leave-one-out cross-validation (LOOCV), and 95% bootstrap confidence intervals (B = 1000 iterations, stratified by diagnostic group). Three models are compared: the full 49,410-probe Elastic Net baseline (Model A), the PyImpetus-selected 6-probe Elastic Net (Model B), and the 6-probe model augmented with seven clinical covariates including APOE4 (Model C). Five-fold CV results are reported for all three models to enable direct comparison on a level playing field. LOOCV results with bootstrap CIs are additionally provided for the six-probe model as the primary near-unbiased performance estimate. nRMSE = normalised RMSE (RMSE divided by the practical outcome range of 12 MMSE points), providing a scale-independent benchmark for cross-study comparison. CI = 95% bootstrap confidence interval. Large R^2^ standard deviations reflect small test-fold sizes (approximately 19 participants per fold) at *n* = 96 and should be interpreted accordingly. Neither five-fold CV nor LOOCV estimates substitute for external validation in an independent longitudinal cohort.

Model	MAE (Mean ± SD) [95% CI Bootstrap]	RMSE|nRMSE (Mean ± SD) [95% CI]	R^2^ (Mean ± SD) [95% CI Bootstrap]
Full Elastic Net (all 49,410 probes)	1.623 ± 0.366	2.113 ± 0.476|nRMSE = 0.176	0.098 ± 0.185
Six-probe Elastic Net (PyImpetus selected)	1.381 ± 0.296 (5-fold) LOOCV: 1.388 [95% CI: 0.981–1.555]	2.003 ± 0.470|nRMSE = 0.167	0.133 ± 0.255 (5-fold) LOOCV: 0.247 [95% CI: 0.089–0.405]
Six-probe + seven clinical covariates	1.417 ± 0.287	2.012 ± 0.476|nRMSE = 0.168	0.133 ± 0.219

**Table 5:** Prediction accuracy by baseline diagnostic group under stratified five-fold CV (mean ± SD). nRMSE = normalised RMSE (RMSE/outcome range = 12 MMSE points). Performance is highest in CN and MCI groups where clinical intervention is most actionable. Reduced accuracy in the AD group reflects greater biological heterogeneity of late-stage cognitive trajectories.

Diagnostic Group	*n*	MAE (Mean ± SD)	RMSE (Mean ± SD)	Clinical Interpretation
Cognitively Normal (CN)	35	0.869 ± 0.302	1.091 ± 0.389	Best accuracy; lowest outcome variance; most relevant for early intervention
Mild Cognitive Impairment (MCI)	32	1.855 ± 0.559	2.263 ± 0.623	Moderate accuracy; high trajectory heterogeneity; primary clinical target group
Alzheimer’s Disease (AD)	29	2.352 ± 0.464	2.907 ± 0.665	Reduced accuracy; highest biological heterogeneity of cognitive trajectories

**Table 6:** Results of the three-model MCP-counter cell-type deconvolution comparison. Model A uses the six probes alone as predictors; Model B replaces them entirely with seven MCP-counter immune cell scores; Model C combines both sets of predictors jointly. Cell-type scores alone are substantially weaker predictors (Model B MAE = 1.503) than the six probes (Model A MAE = 1.271), and adding them to the six-probe model produces only a modest improvement (Model C MAE = 1.232; 3.1% reduction vs. Model A). All six probes survive the joint model with their coefficient signs intact, confirming the signal is transcriptional rather than compositional. MAE = mean absolute error; R^2^ = coefficient of determination; SD omitted as all three models use identical fold assignments.

Model	MAE	R^2^	Interpretation
A: Six probes only (baseline)	1.271	0.385	Discovery baseline; transcriptomic signal only
B: Cell-type scores only (MCP-counter)	1.503	0.124	Substantially weaker—composition does not equal transcription
C: Six probes + cell types (joint model)	1.232	0.437	Marginal gain; all six probes retained with unchanged directional coefficients

**Table 7:** Cross-platform biological validation in AddNeuroMed (GSE63060, *n* = 329). Five probes mapped from Affymetrix HG-U219 to Illumina HumanHT-12 by HGNC gene symbol. *SNX5* significantly replicates (*p* = 0.002) across platforms and continents. *CHD2* significant but reversed (state vs. trajectory distinction described in Discussion) *AQP7* shows no cross-sectional association (r = −0.003, *p* = 0.964), consistent with its role as a trajectory-specific predictor. ANM = AddNeuroMed.

Gene	ADNI-GO Coef.	Expected Direction	ANM Spearman r	*p*-Value	Interpretation
*AQP7*	−0.598	up CN to AD	−0.003	0.964	Longitudinal-specific; flat cross-sectionally-confirms trajectory predictor
*RPS5*	−0.447	up CN to AD	+0.074	0.181	Trend in correct direction; not significant cross-sectionally
*ASS1*	−0.328	up CN to AD	+0.030	0.583	No cross-sectional association detected in AddNeuroMed
*CHD2*	−0.293	up CN to AD	−0.258	<0.001	Significant but reversed-state vs. trajectory distinction (see Discussion)
*SNX5*	+0.441	down CN to AD	−0.170	0.002	Replicated retromer hypothesis validated cross-platform, cross-continent

**Table 8:** CSF biomarker concordance results (UPENNBIOMK, *n* = 43; 44.8% of full cohort; Elecsys immunoassay). Columns show gene or score, expected direction with Aβ42, observed Spearman r, *p*-value, directional consistency, and biological interpretation. All *n* = 43 participants were confirmed amyloid-positive (*Aβ42* < 298 pg/mL). Three of five annotated probes show directionally consistent trends with Aβ42. Composite risk score correlations with p-tau181 (r = +0.133, *p* = 0.394) and total tau (r = +0.076, *p* = 0.627) are additionally reported. Null results reflect insufficient statistical power (*n* = 43; 35% power to detect r = 0.19 at α = 0.05) rather than absence of biological signal.

Gene/Score	Expected vs. AB42	Observed r	*p*-Value	Direction	Interpretation
Risk score (composite)	Negative	+0.194	0.211	No	Underpowered (*n* = 43; 35% power); *n* = 104 needed for 80% power
*AQP7* (harmful)	Negative	−0.186	0.232	Yes	Directional trend consistent with glymphatic–metabolic hypothesis
*RPS5* (harmful)	Negative	+0.193	0.215	No	No association; underpowered; requires larger CSF sub-cohort
*ASS1* (harmful)	Negative	+0.059	0.705	No	No association detected; underpowered
*CHD2* (harmful)	Negative	−0.180	0.249	Yes	Directional trend consistent with chromatin–amyloid interaction
*SNX5* (protective)	Positive	+0.181	0.246	Yes	Directional trend consistent with retromer–amyloid clearance hypothesis

**Table 9:** Visit-stratified external validation results and null model benchmarks. LOO = leave-one-out cross-validation. Null benchmarks provide the minimum performance threshold that any useful predictor must exceed.

Dataset	*n*	MAE	R^2^	Pearson r	Spearman ρ	Note
Training (in-sample)	96	1.275	+0.385	+0.620	+0.654	In-sample training performance
LOO cross-validation	96	1.388	+0.247	+0.509	+0.591	Primary internal validation metric
External—all 12 m	91	1.740	−0.222	+0.016	+0.097	Primary external validation
External—m60 (month 60)	48	1.917	−0.203	−0.013	+0.073	Most comparable to training m48
External—v06 (month 6)	34	1.515	−0.328	+0.050	+0.110	ADNI-2 first screening visit
External—v11 (month 11)	9	1.643	−0.234	+0.368	+0.434	*n* < 10; insufficient for inference
Null: predict training mean	91	1.627	−0.003	—	—	Constant predictor benchmark
Null: predict zero	91	1.571	−0.091	—	—	No-change benchmark

**Table 10:** A direct comparison of PyImpetus-SHAP with three recent blood transcriptomic AD studies across the methodological dimensions that the Background section identified as gaps. The four studies ask genuinely different questions and should not be read as a ranking; the point is simply to show, at a glance, which validation steps have been taken where and what this study does that the others do not. Empty cells indicate the feature was either not reported or not relevant to that study’s design.

Feature	Wang et al. 2024 [15]	Chen et al. 2025 [11]	Meng et al. 2024 [34]	This Study
What was being predicted?	Whether a participant has AD versus normal cognition (cross-sectional)	AD or MCI diagnosis at a single time point	Which UK Biobank participants would progress rapidly (classification)	How much each participant’s MMSE score would change over the next 12 months (continuous, longitudinal)
How features were chosen	SHAP rankings followed by manual curation	Co-expression network modules	Graph neural network learned from population data	PyImpetus Markov Blanket—run four times across two thresholds and two seeds; only probes appearing every time were kept
What the model can explain?	Global SHAP importance scores	Module-level associations with phenotypes	Population-level graph patterns	Exact per-probe SHAP contribution for every individual prediction, with dependence plots showing how each gene’s effect varies across its expression range
Were blood cell proportions checked?	No	No	No	Yes—MCP-counter scores for seven immune populations tested in three models; all six probes retained after correction
Was the signature tested in a second cohort on a different platform?	No	No	No	Yes—five probes mapped by gene symbol into AddNeuroMed (Illumina HumanHT-12, *n* = 329); *SNX5* replicated (*p* = 0.002)
Is there a usable prediction tool?	No	No	No	Yes—Streamlit web app with real-time sliders, risk stratification, and SHAP bar chart; no installation needed
How was performance reported?	Classification AUC	Correlation between modules and cognitive/imaging phenotypes	Population-level classification metrics	LOOCV R^2^ = 0.247, MAE = 1.388; outperforms the full 49,410-probe model by 14.9% in MAE
Is the code available?	Partial	No	No	Full pipeline (Steps 1–7), trained model, and prediction app at https://github.com/SAH-ML/pyimpetus-shap-ad, accessed on 1 June 2026.

## Data Availability

The whole-blood gene expression data used in this study come from the ADNI database, which is openly accessible to registered researchers at https://adni.loni.usc.edu/ (accessed on 1 June 2026) following execution of the ADNI Data Use Agreement [36]. The AddNeuroMed dataset used for cross-platform validation is publicly available without restriction from NCBI GEO (accession GSE63060; https://www.ncbi.nlm.nih.gov/geo/query/acc.cgi?acc=GSE63060, accessed on 1 June 2026) [19]. CSF biomarker data (UPENNBIOMK_MASTER) are available from the same ADNI portal upon registration [45]. The complete analysis pipeline, trained model weights, and the Streamlit real-time prediction tool are freely available at https://github.com/SAH-ML/pyimpetus-shap-ad, accessed on 1 June 2026, with a live interactive demo at https://pyimpetus-shap-ad.streamlit.app, accessed on 1 June 2026; a permanently citable archived version with a Zenodo DOI will be deposited on acceptance. Data collection and sharing for the Alzheimer’s Disease Neuroimaging Initiative (ADNI) is funded by the National Institute on Aging (National Institutes of Health Grant U19AG024904). The grantee organization is the Northern California Institute for Research and Education. In the past, ADNI has also received funding from the National Institute of Biomedical Imaging and Bioengineering, the Canadian Institutes of Health Research, and private sector contributions through the Foundation for the National Institutes of Health (FNIH) including generous contributions from the following: AbbVie; Alzheimer’s Association; Alzheimer’s Drug Discovery Foundation; Araclon Biotech; BioClinica, Inc.; Biogen; Bristol-Myers Squibb Company; CereSpir, Inc.; Cogstate; Eisai Inc.; Elan Pharmaceuticals, Inc.; Eli Lilly and Company; EuroImmun; F. Hoffmann-La Roche Ltd. and its affiliated company Genentech, Inc.; Fujirebio; GE Healthcare; IXICO Ltd.; Janssen Alzheimer Immunotherapy Research & Development, LLC.; Johnson & Johnson Pharmaceutical Research & Development LLC.; Lumosity; Lundbeck; Merck & Co., Inc.; Meso Scale Diagnostics, LLC.; NeuroRx Research; Neurotrack Technologies; Novartis Pharmaceuticals Corporation; Pfizer Inc.; Piramal Imaging; Servier; Takeda Pharmaceutical Company; and Transition Therapeutics.

## Abbreviations

AD: Alzheimer’s disease; ADNI: Alzheimer’s Disease Neuroimaging Initiative; ADNI-GO: ADNI Grand Opportunities phase; APP: Amyloid Precursor Protein; AQP7: Aquaporin 7; ASS1: Arginosuccinate Synthase 1; Aβ42: amyloid-beta 42; BACE1: Beta-site APP Cleaving Enzyme 1; CHD2: Chromodomain Helicase DNA Binding Protein 2; CI: confidence interval; CN: cognitively normal; CSF: cerebrospinal fluid; CV: cross-validation; CDRSB: Clinical Dementia Rating Sum of Boxes; GEO: Gene Expression Omnibus; HGNC: HUGO Gene Nomenclature Committee; lncRNA: long non-coding RNA; LOO: leave one out; LOOCV: leave-one-out cross-validation; MAE: mean absolute error; MB: Markov Blanket; MCI: mild cognitive impairment; MCP-counter: Microenvironment Cell Populations counter; MMSE: Mini Mental State Examination; EN: Elastic Net; p-tau181: phosphorylated tau at threonine 181; REST API: Representational State Transfer Application Programming Interface; RMSE: root mean squared error; RNA: ribonucleic acid; RPS5: Ribosomal Protein S5; SHAP: SHapley Additive exPlanations; SNX5: Sorting Nexin 5; XAI: explainable artificial intelligence.
